# Entre dois mundos: resistência e crise humanitária no Território Yanomami

**DOI:** 10.1590/0102-311XPT258725

**Published:** 2026-05-29

**Authors:** Altair Seabra de Farias

**Affiliations:** 1 Universidade do Estado do Amazonas, Manaus, Brasil.

Os Yanomami são indígenas de recente contato que têm sido impactados drasticamente por uma crise humanitária com origem direta no garimpo ilegal na Terra Indígena Yanomami ^1^. A invasão de garimpeiros gera contaminação por mercúrio ^2^, desmatamento, insegurança alimentar ^3^, violência e a disseminação de doenças ^4^, desestruturando o modo de vida e a saúde do povo Yanomami ^5^. Todos esses fatores, combinados com negligência política e social, agravaram a crise de saúde pública nas últimas décadas.

*Watupari: Um Testemunho da Resistência do Povo Yanomami* ^6^ ultrapassa o estudo etnográfico, se tornando um relato sobre a resistência do povo Yanomami diante das violências que atravessam gerações. Marcos A. Pellegrini - médico e antropólogo - relata sua experiência, com sensibilidade e firmeza: o confronto entre dois universos - o dos Yanomami, profundamente ligados ao território e à cosmovisão da floresta, e o da sociedade externa, caracterizada pela invasão, exploração e omissão do Estado. A obra mistura diário de campo, reflexão antropológica e testemunho político. O título do livro vem de *Watupari*, espírito protetor e guia na cosmologia Yanomami, simbolizando tanto a vulnerabilidade quanto a resistência do povo diante da destruição de seu território.

No capítulo *Histórias sobre o Começo*, o autor apresenta o seu primeiro contato com os Yanomami, descrevendo o processo de adaptação à vida na floresta, aos costumes e às relações sociais. Pellegrini mostra o impacto inicial da diferença cultural, o estranhamento e a progressiva aproximação. Também traz elementos das narrativas míticas Yanomami, integrando o olhar antropológico ao sensível: histórias que explicam a formação do mundo, dos animais, dos rios e dos espíritos.

No capítulo *Sinais do Fim*, é visto o contraste entre o universo Yanomami e as pressões do “mundo de fora”. O autor narra as primeiras evidências das invasões garimpeiras, da devastação ambiental, da chegada de doenças e do medo. Ele mostra como pequenos sinais - rastros nas trilhas, ruídos distantes, comportamentos incomuns dos animais - são interpretados pelos Yanomami como prenúncios de ameaça.

Em seguida, no capítulo *Noite, Fogo, Escuridão* - este é um dos capítulos mais densos da obra, relatando situações de perigo genuíno: ataques, epidemias, mortes e a instabilidade na vida em comunidade. A “escuridão” descrita assume tanto um sentido simbólico quanto literal - noites em alerta, o receio de invasores e a ação dos *xapiri* (espíritos) invocados pelos xamãs para defender a aldeia. O autor observa rituais de cura e de luto e retrata a intensidade emocional das noites Yanomami, nas quais o fogo revela, ao mesmo tempo, a união do grupo e a vulnerabilidade humana diante do caos vindo de fora.

No capítulo *Remédios*, o autor faz uma ponte entre a medicina indígena Yanomami e as práticas da Medicina ocidental. Também aborda a chegada de doenças como malária, gripe e tuberculose, além das dificuldades enfrentadas pelos profissionais de saúde em áreas remotas. O ponto central é o encontro entre saberes: Pellegrini reconhece o valor dos tratamentos e a visão Yanomami, que muitas vezes associa doenças a fatores espirituais, desequilíbrios ou ações de não-humanos, e não apenas a causas biológicas como na Medicina ocidental.

No capítulo *Um dos Napë Pë Era Eu Mesmo* - *napë pë* é empregado para se referir aos não indígenas -, o autor descreve o momento em que deixa de ser reconhecido exclusivamente como “*o médico branco*” e passa a ser considerado um membro integrado ao grupo, estabelecendo vínculos afetivos substanciais. Relata vivências de proximidade, tais como caçadas, deslocamentos fluviais, conversas aprofundadas e a partilha de receios e alegrias. Ressalta, ainda, os aprendizados adquiridos acerca de território, espiritualidade, temporalidade e aspectos da vida, reconhecendo a formação de uma identidade híbrida - um estrangeiro cuja trajetória foi transformada pelo contato intercultural.

No capítulo *Carta para Onde Corre o Rio* - este capítulo funciona como um conjunto de memórias e reflexões escritas “para alguém distante”, talvez para o leitor, talvez para uma versão passada de si mesmo -, o rio funciona como metáfora de fluxo, continuidade e destino. São narradas viagens fluviais, encontros com outras aldeias, momentos de contemplação e episódios de solidão. O autor registra percepções sobre o sofrimento Yanomami diante da destruição ambiental e reflete sobre a responsabilidade histórica de quem testemunha injustiças. No capítulo *Convivência Pacífica*, Pellegrini descreve que a convivência pacífica Yanomami depende de um conjunto de atitudes coletivas, gestos cotidianos e mecanismos culturais de equilíbrio. Diferentemente da sociedade *napë pë*, onde o conflito muitas vezes se transforma em ruptura, sanção ou violência institucional, entre os Yanomami o foco está em evitar que tensões cresçam. O capítulo serve como contraponto à violência dos anteriores: mostra que, apesar de tudo, há uma ética da paz profundamente enraizada entre os Yanomami, e que essa convivência é possível - desde que respeitados.

No capítulo final, projeta-se perspectivas para o futuro. O rio é utilizado como metáfora para representar tanto a trajetória da vida quanto as incertezas enfrentadas pelo povo Yanomami. Pellegrini revisita suas vivências, reconhece perdas ocorridas, destaca manifestações de resistência e enfatiza a necessidade imediata de preservar o território. Trata-se de um capítulo que propõe reflexões sobre o futuro, ressaltando que a sobrevivência dos Yanomami está condicionada às decisões tomadas pelo Brasil. A conclusão do trabalho reforça um apelo ético e político, incentivando a solidariedade, a justiça e o respeito às culturas indígenas.

A ex-ministra da Saúde Nísia Trindade escreveu a contracapa do livro, enfatizando a relevância da obra para a compreensão da resiliência e da força cultural diante de episódios de violência e exploração. Ela aponta a necessidade de promover a construção de um futuro pautado pela justiça e solidariedade, destacando o conceito de convivência “*sob o mesmo céu*”. Outra participação notória é de Júnior Hekurari, liderança Yanomami que confere legitimidade à obra de Pellegrini. A presença dele no prefácio também reforça que o livro é visto pelos Yanomami contemporâneos como algo significativo para a memória, para a resistência cultural e para a denúncia política da situação.

A obra de Pellegrini é recomendada para todos os públicos, entretanto, diferentes leitores terão diferentes experiências de leitura e níveis de compreensão. Nesse sentido, considerando os graus de força e vividez de David Hume ^7^, em que impressões são percepções diretas, mais fortes e vívidas enquanto ideias são cópias enfraquecidas das impressões, derivadas exclusivamente da experiência. Desse modo, leitores que tiveram algum convívio social, profissional, acadêmico com os Yanomami terão impressões vívidas durante a leitura, revisitando suas memórias; enquanto os leitores mais distantes dos Yanomami terão apenas ideias, turvas e menos vívidas do que acontece por lá, mas têm papel importante para convidar a sociedade para conscientização pública e humanitária.

Por fim, o livro destaca a dignidade e a resiliência de um povo que, mesmo enfrentando doenças, fome, devastação ambiental e ameaças constantes dos *napë pë*, continua lutando para preservar seu modo de vida e suas terras. *Watupari: Um Testemunho da Resistência do Povo Yanomami* torna-se assim um documento fundamental, tanto de denúncia quanto de esperança: registra a voz dos Yanomami, seus conhecimentos e sua busca por autonomia, convidando quem lê a compreender que, ao proteger esse povo, também se protege a floresta e a chance de um futuro diferente para todos nós.
